# Flow-Injection Methods in Water Analysis—Recent Developments

**DOI:** 10.3390/molecules27041410

**Published:** 2022-02-19

**Authors:** Marek Trojanowicz, Marta Pyszynska

**Affiliations:** 1 Laboratory of Nuclear Analytical Methods, Institute of Nuclear Chemistry and Technology, Dorodna 16, 02-195 Warsaw, Poland; m.pyszynska@ichtj.waw.pl; 2 Department of Chemistry, University of Warsaw, Pasteura 1, 02-093 Warsaw, Poland

**Keywords:** flow-injection analysis, water analysis, microfluidics, portable instruments, spectrophotometry, electrochemical detections, ICP-MS

## Abstract

Widespread demand for the analysis and control of water quality and supply for human activity and ecosystem sustainability has necessitated the continuous improvement of water analysis methods in terms of their reliability, efficiency, and costs. To satisfy these requirements, flow-injection analysis using different detection methods has successfully been developed in recent decades. This review, based on about 100 original research papers, presents the achievements in this field over the past ten years. Various methodologies for establishing flow-injection measurements are reviewed, together with microfluidics and portable systems. The developed applications mostly concern not only the determination of inorganic analytes but also the speciation analysis of different elements, and the determination of several total indices of water quality. Examples of the determination of organic residues (e.g., pesticides, phenolic compounds, and surfactants) in natural surface waters, seawater, groundwater, and drinking water have also been identified. Usually, changes in the format of manual procedures for flow-injection determination results in the improvement of various operational parameters, such as the limits of detection, the sampling rate, or selectivity in different matrices.

## 1. Introduction

The protection of natural water resources on a global scale is one of the most significant challenges of civilization at the beginning of the third decade of the 21st century. Escalating climatic change is the largest contributor to the greenhouse effect, connected with the alteration of atmospheric composition and the increasing emission of anthropogenic pollutants into the environment, which contribute to a reduction in the available volume of fresh natural waters and the lowering of the below-ground water table. They are also responsible for biological degradation and the reduction in chemical quality of the whole aquatic environment, including surface water, groundwater, and the seas and oceans. This key problem for modern civilization is currently the object of very intensive biological, chemical and physical scientific investigation, as well as numerous initiatives and research programs in the fields of environmental engineering and the design of measuring instruments, and systems for monitoring environmental changes [1]. This also includes the search for legislative and technological solutions, in order to stop or reduce those catastrophic environmental changes.

One of the most significant factors affecting the current status of natural waters, which are very difficult to fully control and for which limitations need to be introduced, is the emission of anthropogenic pollutants into the environment. Although in different proportions, this problem concerns the behavior of individual members of the population as well as on a much larger scale, in terms of the processes associated not only with different branches of industry but also with agriculture, transport, and the mining of fossil-fuel materials and minerals. In the middle of the previous century, the main identified anthropogenic pollutants of natural waters were heavy metals ions, the residues of pesticides, and some groups of organic compounds of industrial origin, including primarily the so-called persistent organic pollutants (POPs), such as organic solvents or detergents. Now, the list of these anthropogenic chemical environmental pollutants is much longer, which is confirmed by the very extensive literature in the fields of environmental chemistry and engineering, toxicology, and health protection. Since the 1970s, particular attention has been focused on endocrine-disrupting compounds (EDCs). Apart from pesticides, these include not only plasticizers and phenolic compounds but also such emerging contaminants as human and veterinary drugs, and certain ingredients in personal care products and cosmetics [2,3]. The main reason for this interest is the fact that certain EDCs, for instance, steroids, nonsteroidal antibiotics, analgesics, and anti-inflammatories or anti-depressants, may induce physiological effects in humans, even at very low doses [4]. About 160 human and veterinary pharmaceutical products and about 30 by-products are considered to carry this risk, spread over 24 therapeutic classes [5]. The main source of their presence in the environment, and hence in drinking water, for example, is the discharge of municipal wastewater effluents [6]. Another group of organic anthropogenic environmental pollutants on which particular attention has been focused since the early 2000s is the poly- and perfluoroalkyl substances (PFASs). Due to their exceptional chemical stability and numerous commercial applications, they frequently occur in an aqueous environment [7]. Wastewater treatment plants are considered likely to be the main contributor to their presence in the surface waters and, hence, also in drinking water [8], as well as industrial sites, military firing ranges and training areas, and civilian airports [9].

In the past two decades, completely different anthropogenic pollutants, such as microplastics and nanomaterials, are pointed out as especially problematic in aquatic systems. The frequent discarding of plastics into natural water bodies, followed by degradation via mechanical and photochemical fragmentation, results in the formation of polymeric microparticles that may remain in the environment for hundreds of years, additionally accumulating other chemical pollutants [10]. The increasing importance of nanotechnology on an industrial scale means that engineered nanoparticles are commonly detected in natural water bodies [11]. Their high surface-to-volume ratio and outstanding reactivity are the sources of their highly dynamic transformations in the environment [12]. Special attention is also being paid to the presence in the aquatic environment of silver nanoparticles [13], and the transformation and bioavailability of metal oxide nanoparticles [14]. The latter, due to their electronic and magnetic properties, are widely employed as components in different commercial products (e.g., polymers and catalysts), finding numerous environmental applications (sensing, remediation); they are also employed in health care as antimicrobials and in cancer treatment.

Monitoring the aquatic environment and applying efficient methods for its protection is impossible without employing adequate chemical analytical methods. Besides the adaptation of analytical methods for laboratory, field, or process-control use, they should fulfill general criteria regarding the quality of chemical analysis. These include suitability for a given range of analyte concentrations, selectivity, the required precision of determination, and accuracy. In the present state of development in analytical chemistry, an exceptionally rich arsenal of measuring instruments and developed analytical methods is available for the analysis of water bodies and wastewaters of different origins. They also allow the virtual monitoring of each type of identified pollutant. Certain limitations to their practical use may be due to economic reasons in particular circumstances or to the availability of sufficiently trained personnel.

The literature available on water analysis is very wide-ranging and includes numerous dedicated books, original research articles, and reviews in scientific and technical journals, as well as the standard procedures reported in the regulations of various organizations, such as ISO, EPA, etc. Periodic reviews on the newest achievements in this field are published, e.g., in the ACS journal, *Analytical Chemistry* [15]. Determination of the trace content of organic micropollutants in samples with complex matrices is particularly challenging, for which high-resolution mass spectrometry and multidimensional chromatography are especially effective [16], as well as systems hyphenating high-performance liquid chromatography or gas chromatography instruments with mass spectrometry or nuclear magnetic resonance [17]. A particular difficulty in the trace determination of, e.g., emerging persistent organic pollutants is the development of efficient methods for the isolation and preconcentration of analytes [18,19]. In recent decades, one very favorable element of the scaling-down of measuring instrumentation, including their applications in water analysis, is the development of sensor technology. Generally speaking, this includes reagent-free measuring devices, which must be low-maintenance and autonomous; these are specific for a given analyte or group of analytes with the use of the appropriate detection technique [20]. Good examples of such devices that are reported in the literature and commercially available sensors and biosensors include semiconductor-based heavy metal ion sensors [21], membrane ion-selective electrodes, as well as biosensors based on the use of enzymes, antibodies, DNA, aptamers, and other synthetic biology tools [22]. New challenges in this field include the development of efficient methods for the determination of nanoparticles in the environment [23,24] and identifying microplastics [25]. A separate function, also contributing to water quality control, is microbial water analysis [26].

## 2. Flow Chemistry in Contemporary Chemical Analysis

Because of a widespread need for the analysis of waters and wastewaters and for monitoring of the aqueous environment, the miniaturization of measuring devices plays quite an essential role. This is manifested by the increasing application of integrated sensors and biosensors, as well as the design of flow analyzers [27]. Measuring devices for the continuous monitoring of aqueous ecosystems also need to take into account the rules of green analytical chemistry in their design as much as is technologically possible, particularly in terms of the limitation of reagent consumption and the generation of waste [28]. In this context, flow injection analysis (FIA) methods are considered to be especially suitable [29].

Flow chemistry is an increasingly recognized and exploited field of chemical science, in which the hydrodynamic properties of flowing liquids are utilized for conducting chemical reactions. This concerns the conducting of reactions for synthetic purposes (unfortunately, in this case, given the commonly but rather improperly used term “flow chemistry”), for conducting analytical determinations of flow conditions, for carrying out basic kinetic investigations, and also for performing industrial technological processes [30]. In all these fields, the flow conditions are utilized for regulating the transport of reagents, improving interphase contacts, for the enhancement of heat transfer, and for the safe manipulating of hazardous substances.

Pioneering steps in flow synthesis have been associated with the use of flow-through reactors (see, e.g., [31]). Regarding the first analytical applications of flow chemistry, one can refer to much earlier works on column chromatography [32,33,34]. Although they were, undoubtedly, analytical works, the pioneering studies by Skeggs on the development of “flow analysis” in the middle of the 1950s are well respected [35]. One bewildering situation must be acknowledged: even in very competent reviews, published in the most prestigious journals (see, e.g., [36]), one can find the opinion that “continuous flow has affected many fields over the last 20 years” without there being any acknowledgment that analytical applications have been developed for almost 70 years, in thousands of publications, and in widely and routinely used procedures. In those pioneering works by Skeggs, the essential instrumental novelty was the development of multistep analytical procedures for photometric determination. These were carried out under continuous-flow conditions, via the configuration of different flow-through modules, for example, for mixing solutions, incubation, dialysis, and detection. This method of mechanizing analytical determinations resulted in an evident improvement of the efficiency of determination, and in significant enhancement of the precision of analyses. This concept has been widely accepted, first in analytical clinical laboratories, and very soon after in environmental, agricultural, and industrial ones [37].

**Figure 1:**
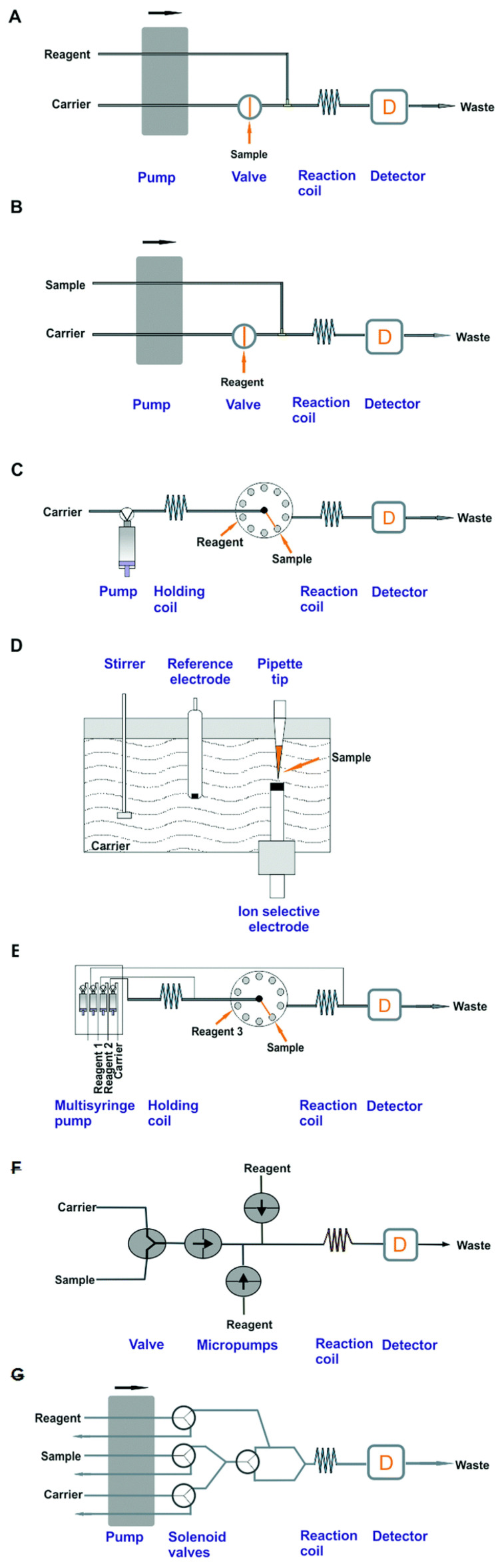
Schemes of the manifolds of basic flow injection systems [45]: (**A**)—normal flow injection analysis (FIA) system, (**B**)—reverse flow injection analysis (rFIA) system, (**C**)—sequential injection analysis (SIA) system, (**D**)—batch injection analysis (BIA) system, (**E**)—multisyringe flow injection analysis (MSFIA) system, (**F**)—multicommutated flow analysis with a multichannel pump system, (**G**)—multi-pumping flow analysis system. Reproduced with permission from *Analyst*; published by the Royal Society of Chemistry, 2016.

As a further essential improvement of that analytical methodology, which resulted in the reduction of the required sample volume, the decrease in consumption of reagents, and the shortening of measurement time, the invention of flow injection analysis is commonly recognized as significant. At the beginning of the 1970s, several research groups [38,39,40,41,42] invented the concept of flow analysis, with the injection of a small sample volume into a flowing stream of carrier solution. The term “flow-injection analysis” was introduced by Ruzicka and Hansen in a series of ten papers published in the journal *Analytica Chimica Acta* in 1975–1978; since then, it has been widely accepted by the analytical community. A further instrumental improvement of the flow-injection methodology was brought by the concept of sequential-injection analysis (SIA), mostly due to an even better cost-efficiency of determinations than that usually achieved by FIA systems [43]. In SIA systems, small segments of the sample and reagent(s) are introduced into the holding coil, which is then followed by pumping them in the opposite direction toward the detector. During this process, the partial overlapping of segments usually takes place, which allows the reaction to occur between the analyte and the reagent. The significant interest of analysts in flow analytical methods and the development of their numerous practical applications over six decades has resulted in the design of many types of flow analytical systems [44]; examples of those that are most commonly used are given in Figure 1.

The methodology of flow-injection measurements and their different variants have a very solid place in modern analytical chemistry [45]. It is the subject of thousands of original research papers and numerous books, and the apparatus for performing it is commercially available; however, it can be assembled relatively simply from parts and accessories usually found in chemical research laboratories. The main attributes of those methods are the small sample volume needed (usually 50 to 100 μL), detection during the flow of the sample solution through a detector, and the conducting of possibly all required sample processing operations in a properly designed flow-through module.

In such cases, virtually all analytical techniques of detection that are available for taking measurements in solutions can be used, although the largest number of applications were developed for UV-Vis absorption spectrophotometry (see. e.g., review [46]). Such systems are usually constructed for the determination of single analytes, but the setups for flow multicomponent determination can also be designed in different ways. This can be achieved by a smart extension of the flow manifold with various detectors, or via the application of multicomponent detectors, as well as by suitable data processing using advanced chemometric methods (see, e.g., [47]). One example of such an approach is the so-called multicommuted system, where, with the application of a combination of single-channel solenoid micro-pumps and valves, or by the use of a multichannel pump with several solenoid valves (see Figure 1F,G), one can achieve time-based control of the sample injection and merging of segments of solutions [48]. In order to reduce additionally the consumption of reagents, a configuration of reverse flow-injection analysis can be employed, whereby a very small volume of reagent (usually also 50 to 100 μL) is injected into the setup along with a continuous flow of aspirated samples (Figure 1B) (see, e.g., review [49]). A very wide application of the FIA and SIA concepts is also reported in numerous papers presenting multisyringe pumping systems (Figure 1E) [45,50]. In such a configuration, the flow of solutions in a single or multichannel setup is propelled by the assembly of mechanically controlled syringes as, used commonly, for instance, in mechanized titrators. Each syringe head is equipped with a solenoid-driven valve to direct the solution flow. The scheme shown in Figure 1D depicts the principle of a so-called batch injection analysis (BIA) system, where the sample to be analyzed is injected directly onto the sensing surface of the detector. This provides a very fast response, but the possibility of on-line sample processing is very limited in such a case.

One very frequent misunderstanding is in calling flow analytical systems automated systems. In reality, this term should only be used in the situation when the employed computerized control system is able, with the use of a feedback loop in the software, to optimize the operating conditions of sample determination without the intervention of a human operator. It must be admitted that, in fact, in recent years there have been some examples of such smart systems (see, e.g., the recent review by [51]).

One important development trend in flow-injection instrumentation that has been observed in the past few decades is the miniaturization and integration of different components and modules of the measuring setup into a single instrument. This was initiated firstly by the development of integrated micro conduits [52], then, the design of multifunctional injection valves, the so-called lab-on-valve (LOV) with incorporated miniaturized detectors or microcolumns packed with solid sorbents [53]. A valuable innovation in this trend, from the point of view of different applications, including water analysis, is the adaptation of a design for such systems of a microfluidic device, with flow-through channels that are usually of a few tens of micrometers. This trend was begun at the end of the previous century (e.g., [54]), and then additionally enhanced by progress in nanotechnology [55], as well as by technology for their preparation in paper matrices [56].

## 3. Flow-Injection Methods in Water Analysis

Among the different applications of flow-injection analysis, their applications in water analysis are particularly broad. The method is favored first of all because of the wide demand for carrying out water quality control analyses for municipal needs, for industrial monitoring, and also for food production. In the first two decades of the development of laboratory flow analytical methods, with the segmentation of flowing streams and the use of the steady-state detector response as an analytical signal, their main field of application has been in clinical analysis. However, soon after, environmental applications have appeared, primarily in water analysis. The method’s medical applications were replaced in subsequent decades by the more efficient discrete analyzers, while from the middle of the 1970s, numerous applications of FIA methods have already focused on the analysis of waters of various origins.

Natural waters present a relatively simple type of matrix for the conducting of fast-flow analysis determinations since, except for seawater, they are not heavily loaded matrices requiring complex sample processing prior to performing the detection operation. The main advantages of using the various FIA methods include the mechanization of analytical procedures, leading to more efficient analyses and improvements in the precision of determination. The possibility of conducting the necessary sample processing in on-line mode, which results in the simplification of the whole multistep procedure, is especially attractive. The application of appropriate operations when isolating the analyte(s) from the sample matrix or the inclusion of a preconcentration step results in an enhancement of the selectivity of determination and an improvement in the limit of detection (LOD). Properly designed FIA systems can be fully integrated and mechanized complete analyzers or can serve as accessories for preliminary sample processing, prior to chromatographic, capillary electrophoretic, or mass spectrometry analyses.

A comprehensive description of the development of FIA methods for water analysis can be found in numerous books, dozens of review articles in scientific journals, and in the very rich literature of the original research works. The subject of this review is the presentation of development trends in this area of FIA applications in the past decade. Besides presenting the details of numerous original research works, it seems to be appropriate to mention several review papers that have been published on this subject since 2010 (Table 1). To some extent, these articles indicate the selection of the determined analytes, describe the detection methods employed, and show the configurations of the flow-injection systems developed. Among the analytes that have been determined, one can find trace amounts of numerous organic compounds [57,58], the residues of pharmaceuticals [59], and also many groups of inorganic analytes [60,61,62], including nutrients in oceanographic measurements [63], as well as the environmentally significant radionuclides [64,65,66]. Flow-injection analysis methods are also employed in the determination of the total indices of water quality [57,58,59,60,61,62,63,64,65,66,67]. Due to their usually rapid determinations, they are effective in the on-line monitoring of drinking water quality and can be employed as early warning analytical systems [68]. The recently published reviews also demonstrate one important role of the microfluidic systems developed for water analysis [69,70], and also, in recent years, microfluidic paper-based analytical devices [71], which offer particular advantages that, besides the miniaturization of measuring setups, should also include the low cost of their mass production.

**Table 1:** Review articles published in the past decade in scientific journals on the application of flow methods in water analysis.

Subject	1st Author, Year of Publication	Reference
Automation of radiochemical analysis by applying flow techniques to environmental samples	Fajardo, 2010	[64]
Flow analysis techniques as effective tools for the improved environmental analysis of organic compounds, expressed as total indices	Maya, 2010	[67]
Advances in on-line drinking water-quality monitoring and early warning systems	Storey, 2010	[68]
Flow-based methods with chemiluminescence detection for food and environmental analysis	Christodouleas, 2011	[60]
Determination of total organic fluorine (TOF) in environmental samples, using flow-injection and chromatographic methods	Musijowski, 2011	[57]
Advances in microfluidics for environmental analysis	Jokerst, 2012	[69]
Flow-injection analysis as a tool for the determination of pharmaceutical residues in an aqueous environment	Trojanowicz, 2012	[59]
Flow injection analysis as a tool for enhancing oceanographic nutrient measurements	Worsfold, 2013	[63]
Application of microfluidics in waterborne pathogen monitoring	Bridle, 2014	[70]
Application of flow analysis in the determination of selected radionuclides	Kołacińska, 2014	[65]
Analytical challenges and advantages of using flow-based methodologies for ammonia determination in estuarine and marine waters	Sraj, 2014	[61]
Developments of microfluidic paper-based analytical devices (μPADs) for water analysis	Almeida, 2018	[71]
Preconcentration of organic substances on low-polar adsorbents in the flow systems of analysis	Tsizin, 2018	[58]
Dynamic flow, considered for automated radiochemical analysis in environmental, nuclear and medical applications	Qiao, 2020	[66]
Development of testing methods for water quality using flow analysis	Teshima, 2020	[62]

### 3.1. Flow-Injection Systems with Spectroscopic Detection

In all the above-mentioned detection techniques employed in flow-injection analysis, the spectroscopic methods, both molecular and atomic spectroscopy, are the most widely used. This is particularly the case in the application of FIA methods to the analysis of natural waters and waters used after treatment for various purposes. As those applications have been developed over about half a century, they have gone through very different instrumental and methodological evolutions.

Without any doubt, the most commonly used detection methods are absorptive spectrophotometry and luminescence detections in the visible range of radiation; the UV or infrared range is much less frequently employed. This is also reflected in works published in the past decade [72,73,74,75,76,77,78,79,80,81,82,83,84,85,86,87,88,89,90,91,92,93,94,95,96,97,98,99,100,101,102,103,104,105,106,107,108,109,110,111,112,113,114,115,116], as can be seen in the data collected from the original research works in Table 2. The great popularity of these methods is generally the result of their wide use in analysis, not only in flow methods, because of their simplicity, the broad availability of the necessary apparatus and reagents, and the large number of already developed non-flow methods. Obviously, these recent developments are directed toward the improvement of detectability and selectivity, achieving a shorter time for analysis, the miniaturization of instrumentation, and the mechanization or automation of the whole procedure. Some basic information on recently developed procedures is given in Table 2.

In the determination of many analytes in natural waters, one crucial parameter, in many cases, is the possibility of achieving a low limit of detection (LOD). A simple instrumental tool to achieve this is to use long-path flow cells in the absorptive flow measurements [72,73]. In the determination of ammonia in seawater, the obtained LOD was at a nanomolar level [72], while in the case of nitrite and nitrate, it was even at a sub-nM level [73]. One especially attractive property of FIA systems is the possibility of carrying out different operations of sample processing in the flow mode, which practice is very widely used for the improvement of the detectability and selectivity of determinations. In the SIA-LOV system, a solid-phase extraction (SPE) with renewable beads was employed for the determination of the residues of chlorotriazine herbicides in ground- and tap water at a sub-μg L^−1^ level [74]. With the use of a 3D-printed disc module, SPE was employed for the trace analysis of Cr(VI) [75]. Then, in the SIA-LOV setup, the application of a complex formation on resin beads allowed researchers to obtain a sub-μg L^−1^ LOD in the determination of Pb(II) in both natural and tap water [76].

**Figure 2:**
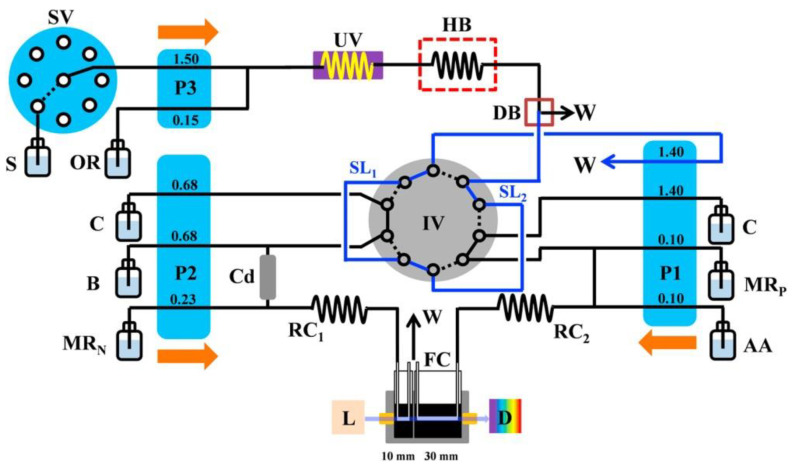
Schematic diagram of an FIA system developed for the determination of the total dissolved nitrogen and phosphorus, with photometric detection and on-line UV and thermal digestion [88]. S—samples or standards; C—ultrapure water carrier stream; B—ammonium chloride buffer; OR—oxidation reagent; MR_N_—mixing reagent for nitrogen determination; MR—mixing reagent for phosphorus determination; AA—ascorbic acid; Cd—cadmium column; P1–P3—peristaltic pumps; IV—10-port injection valve; SV—8-position selector valve; DB—de-bubbler; L—light source; D—detector; FC—“U” shape flow cell; UV -UV-digester; HB—heated bath; RC—reaction coil; SL—sample loop; W—waste. The dashed line in IV represents the valve in position A, and the solid line represents the valve in position B. Reproduced with permission from *Talanta*, published by Elsevier, 2018.

Particularly low levels of LOD (ng L^−1^) were reported in the determination of phosphate with preconcentration on a polymer inclusion membrane [77], as well as by coprecipitation in the determination of U(VI) in seawater [78]. Among other novel methods of on-line preconcentration of analytes in FIA systems with spectrophotometric detection, it is worth mentioning a smart in-syringe dispersive liquid-liquid extraction method for analyte isolation and enrichment [79], as well as magnetic-stirring-assisted dispersive liquid-liquid extraction [80]. Generally speaking, dispersive liquid-liquid extraction is a separation process that is based on the dispersion of fine droplets of the extracting solvent in an aqueous sample. Another relatively novel separation process employed for analytical purposes is pervaporation. This is a membrane-based process for the separation of liquid mixtures by partial vaporization, using non-porous membranes made of polymeric or ceramic materials. On-line pervaporation has been employed, for instance, for the elimination of turbidity and organic interference in the speciation analysis of arsenic in river water [81]. Several other spectrophotometric methods were also developed in the FIA and SIA systems [82,83,84,85,86]. Among general parameters characterizing the water quality, a novel attempt was reported regarding the FIA determination of total dissolved nitrogen, with the use of on-line UV digestion and reduction by VCl_5_ with an LOD of 40 μg L^−1^ [87]. The FIA method was also reported for the simultaneous determination of total dissolved nitrogen (TDN) and total dissolved phosphorus (TDP) [88]. As is shown in Figure 2, the developed system was equipped with one spectrophotometric detector, with two optical paths and on-line UV and thermal digestion. This is an especially interesting example of a smart system for a flow-injection procedure involving two complex on-line operations for sample processing. The reported sampling rate, of about 5 h^−1^, makes this setup particularly attractive for routine application.

The works listed in Table 2 indicate that FIA methods with spectrophotometric detection are much more frequently used for the determination of inorganic analytes than organic ones, due to the better selectivity of the reactions of inorganic analytes with color-forming reagents.

**Figure 3:**
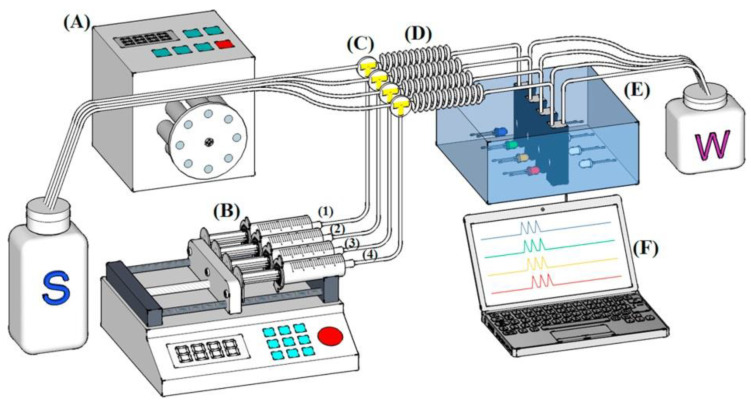
Schematic diagram of a multicomponent reversed-FIA system, with LED-based photometric multi-optical detection for the determination of transition metal ions in river water [89]. A—peristaltic pump; B—multisyringe pump (consisting of four syringes containing color-forming reagents; C—solenoid valves; D—mixing coil; E—multi-optical sensor and F—computer; S-sample; W-waste; (1),(2),(3),(4)—reagents for each analyte. Reproduced with permission from *Talanta*, published by Elsevier, 2017.

Systems developed for the multianalyte determinations seem to be especially attractive for routine applications, as also shown in Table 2. Besides the abovementioned system with one detector [88], another interesting setup is a reverse-FIA system for the determination of transient metal ions with LED-based multi-optical detection, which is depicted schematically in Figure 3 [89]. The use of a purpose-built multi-channel photometer allowed the researchers to obtain a very satisfactory efficiency of determination (4 analytes × 15 samples per h).

This methodology, which can also be employed for simultaneous determination under flow conditions, is called gradient titration. Such a system was reported, e.g., for the simultaneous determination of calcium and magnesium, with an LOD at sub-mg L^−1^ level, based on the use of complexometric titration in the SIA system with photometric detection [90]. It should be mentioned that the most common manual titrimetric methods with different detections are employed for millimolar rather than sub-mg L^−1^ concentrations of the analytes (except for some special techniques of microtitration). Very few reported determinations of organic analytes are carried out, mostly in FIA or SIA systems hyphenated to high-performance chromatographic separations [74,79,80,91].

**Table 2:** Application of molecular spectroscopy detection methods in flow-injection systems for water analysis.

Analyte(s)	Type of Water	Detection Method	Type of Flow System	Employed Sample Processing/Remarks	LOD, mg L^−1^	1st Author, Year of Publ.	Reference
Ag(I)	Drinking water	TLS	FIA	Determination based on the formation of colloidal silver nanoparticles, via reduction with NaBH_4_	0.0015	Korte, 2011	[99,103]
Ammonia	Surface, sea, and tap waters	Fluoresc.	SIA	SIA system with pressure-assisted dual-headspace gas-liquid microextraction module for membraneless gas separation	50 ng L^−1^	Giakisikli, 2018	[93]
Ammonium	Sea water	UV/Vis abs.	FIA	FIA system with on-line reaction in a knitted, heated reaction coil and long-path cell	3.5 nM	Zhu, 2014	[72]
Ammonium	River waters	UV/Vis abs.	Reverse FIA	-	70 nM	Lin, 2018	[100]
Al(III)	Surface and tap waters	UV/Vis abs.	Flow-batch system	Sequential injection-mono-segmented flow system incorporating a mixing chamber	0.020	Khanhua- thon, 2015	[101]
Al(III), Cr(V)	Surface waters	UV/Vis abs.	Flow-batch system	Multi-commutation system, with a stop cell equipped with a web camera for digital imaging	Al: 0.00397 Cr: 0.00265	Andrade, 2013	[102]
As speciation (inorganic)	River water	UV/Vis abs.	SIA	On-line pervaporation cell employed for the elimination of turbidity and organics	As(III): 0.022 As(V): 0.051	Boonjob, 2013	[81]
Ca and Mg	Surface waters	UV/Vis abs.	SIA	Simultaneous determination using flow-injection gradient titration	Ca 0.3 Mg 0.1	Kozak, 2018	[90]
Cd(II) and Pb(II)	Surface and well waters	Fluoresc.	LOV-SIA	On-line SPE for separation and preconcentration	Cd 0.0002 Pb 0.00017	Mattio, 2018	[94]
Chlorotri-azine herbicides	Ground and tap	UV/Vis abs.	LOV-SIA- HPLC	SPE separation and preconcentration of analytes in LOV-SIA system and renewable beads	LOQ: 0.07–0.12 μg L^−1^	Boonjob, 2010	[74]
Co(II)	Well waters	UV/Vis abs.	Multisyringe FIA	Kinetic-catalytic determination with microconduit chip for efficient mixing	20 ng L^−1^	Abouhiat, 2017	[104]
COD	River and waste-waters	Chemlum.	FIA	-	0.083	Hue, 2017	[97]
Cr(III), Cr(VI)	River water	UV/Vis abs.	FIA	Micropumping multicommutated system with LED detector	Cr(III) 2.05 Cr(VI) 1.0 g L^−1^	Pires, 2015	[82]
Cr(VI)	Surface and ground- water	UV/Vis abs.	Multisyringe FIA	On-line SPE preconcentration using a 3D-printed disc-based module	0.0005	Calderilla, 2018	[75]
Cu(II)	River water	UV/Vis abs.	LOV-SIA	SPE with renewable micro-beads	0.003	Yu, 2012	[105]
Cu	River and waste water	UV/VIS abs.	LOV-SIA	Catalytic method with SIA system, employing a micronduit for solution mixing	0.00012	Phansi, 2014	[117]
Cu and Fe	River waters	UV/Vis abs.	LOV-SIA	-	Cu 0.018 Fe 0.015	Gonzalez, 2017	[106]
Fe	Sea water	UV/Vis abs.	LOV-FIA	Procedure with stopped flow in a holding coil or flow-through cuvette	0.00057	Hatta, 2018	[107]
Fe(II), Fe(III)	Artesian water	UV/Vis abs.	SIA	Single peak procedure for the simultaneous determination of analytes	Fe(II) 0.04 Fe(III) 0.09	Kozak, 2016	[83]
Fe(II), Fe(III)	N.a.	UV/Vis abs.	FIA-IC	Low-pressure chromatography with a post-column reaction and derivatization for detection	Fe(II) 1.55 Fe(III) 3.09 μg L^−1^	Chen, 2015	[84]
Fluoride	Tap water	UV/Vis abs.	FIA	On-line SPE preconcentration using a mini-column packed with layered double hydroxide sorbent	0.015	Rocha, 2018	[118]
Fluoroqui- nolones	River water	Fluoresc.	FIA-HPLC	On-line SPE in FIA system hyphenated to HPLC for the determination of norfloxacin, ciprofloxacin and enrofloxacin	6–19 ng L^−1^	Peixoto, 2018	[91]
Hg(II)	Lake, river, tap	Chemlum	FIA	Immunoassay based on the use of resin beads, enzymatic amplification, and a novel monoclonal antibody	15 ng L^−1^	Xu, 2015	[98]
Nitrate	Tap, surface, waste-waters	UV/Vis abs.	FIA	Detection with the use of paired emitter-detector diodes	0.00073	Cogan, 2013	[108]
Nitrite, nitrate	Seawater	UV/Vis abs.	Multisyringe LOV-SIA	Extended typical LOV by a chip, integrating reaction and mixing channels	NO_2_^−^ 30 nM NO_3_^−^ 100 nM	Horstkotte, 2013	[85]
Nitrite, nitrate	Seawater	UV/Vis abs.	Reverse FIA	Detection with a long path-length waveguide capillary cell	Both analytes 0.6 nM	Feng, 2013	[73]
Nitrite, nitrate, phosphate, silicate	Seawater	UV/Vis abs.	LOV-SIA	Determinations based on a single, salinity-independent calibration, with standards prepared in distilled water	NO_2_^−^ 12 nM NO_3_^−^ 94 nM PO_4_^3−^ 47 nM SiO_3_^2−^ 0.24 μM	Hatta, 2021	[109]
Nitrite, phosphate, silicate	Artificial seawater	UV/Vis abs.	CFA	Flow system with a bubble-free flow cell	NO_2_^−^ 60 nM PO_4_^3−^ 80 nM SiO_3_^2−^ 110 nM	Lin, 2017	[110]
Nitrite, nitrate, phosphate, Mn, Fe(II), Fe(III)	Surface and tap waters	UV/Vis abs.	Reverse FIA	Sequential determinations without sample processing	0.03–0.7 μM	Lin, 2017	[86]
Nitrogen, total dissolved	Mineral, tap and surface	UV/Vis abs.	FIA	On-line UV digestion and reduction by VCl_3_	0.04 mg N L^−1^	Lin, 2021	[87]
Parabens	Tap- and seawater	UV	SIA-HPLC	SIA system with on-line in-syringe dispersive liquid-liquid extraction and robotic phase separator	0.0003 to 0.0013	Medina, 2018	[79]
Paraquat	River, tap, rice field water	UV/Vis abs.	FIA	Off-line preconcentration on cation exchanger	0.15	Chuntib, 2015	[111]
Pb(II)	River waters	UV/Vis abs.	CFA	3D-printed resin column for a sorbent, mixing coil and detection cell	0.0027	Mattio, 2017	[112]
Pb(II)	Ground, tap, well waters	UV/Vis abs.	Multisyringe LOV-SIA	On-line SPE of analyte, followed by complex formation on resin beads	0.00079	Rodriguez- Maese, 2020	[76]
Phenolic compounds	Mineral, well, tap	UV/Vis abs.	LOV-SIA- HPLC	On-line magnetic-stirring-assisted dispersive liquid-liquid micro-extraction before HPLC	0.04 to 0.46	Gonzalez, 2015	[80]
Phosphate	Seawater	UV/Vis abs.	Reverse FIA	-	50 ng L^−1^	Li, 2012	[119]
Phosphate	Mineral, melted snow	UV/Vis abs.	FIA	Separation and preconcentration of an analyte with the use of a polymer inclusion membrane	40 ng L^−1^ PO_4_-P	Nagul, 2013	[77]
Phosphate	Synthetic seawater	Fluoresc.	Reverse FIA	Fluorescence excited via an axially coupled fiber providing LED light and emission detected by photodiode	0.00045 PO_4_-P	Kröckel, 2014	[95]
Phosphate and silicate	Ground and river waters	UV/Vis abs.	FIA	Simultaneous determination based on single peak recording	PO_4_: 0.054 SiO_3_: 0.092	Kozak, 2015	[113]
Sulfide	Hot spring waters	Fluorescence	FIA	System with on-line membrane-based micro-channel device for the extraction of free sulfides	0.05 μM	Toda, 2012	[96]
Surfactants, cationic	Mineral, tap, well	UV/Vis abs.	Multisyringe FIA	On-line in-syringe dispersive liquid-liquid microextraction with solvent washing	<30 nM	Horstkotte, 2014	[114]
TDN, TDP	River water	UV/Vis abs.	FIA	On-line UV and thermal digestion and reactions, leading to colored products	TDN 0.8 μM TDP 0.2 μM	Lin, 2018	[88]
Th and U	Surface and tap waters	UV/Vis abs.	Multisyringe LOV-SIA	On-line SPE separation and preconcentration	Th 60 ng L^−1^ U 5.9 ng L^−1^	Avivar, 2011	[115]
Transition metal ions Cu, Fe(II), Zn	Mineral, river, tap	UV/Vis abs.	SIC (FIA-HPLC)	Post-column derivatization for UV/Vis abs. detection	0.6–2.9 μM L^−1^	Horstkotte, 2015	[92]
Transition metal ions Cu, Mn, Fe	River water	UV/Vis abs.	Reverse FIA	Multicomponent reversed FIA system with LED-based multi-optical detection	0.011–0.050	Youngvises, 2017	[89]
U(VI)	Seawater	UV/Vis abs.	FIA	On-line preconcentration by co-precipitation	10 ng L^−1^	Kuznetsov, 2014	[78]
U(VI)	Ground waters	UV/Vis abs.	Multisyringe FIA	-	0.3	Danchana, 2019	[116]

Abbreviations used: CFA—continuous flow analysis system, COD—chemical oxygen demand, ET–AAS—electrothermal atomic absorption spectrometry, FIA—flow injection analysis system, IC—ion-chromatography, LOV—lab-on-valve, SIA—sequential injection analysis system, SIC—sequential injection chromatography, SPE—solid-phase extraction, TDN—total dissolved nitrogen, TDP—total dissolved phosphorus, TLS—thermal lens spectrometry, UV/Vis abs.—UV/Vis absorption spectrophotometry.

**Figure 4:**
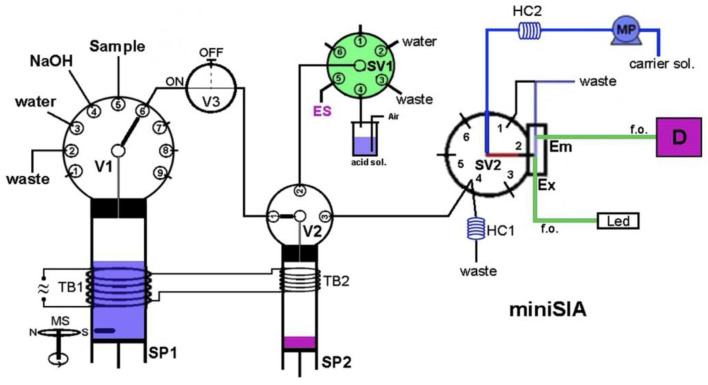
Schematic diagram of an SIA system developed for the determination of ammonia in waters with fluorimetric detection and a pressure-assisted dual-headspace gas-liquid micro-extraction module for membraneless gas separation [93]. SV—selection valve; V—valve; MS—magnetic stirrer; SP—syringe pump; MP—pump; TB—thermostat barrel; HC—holding coil; Ex—excitation; Em—emission; D—detector; W—waste. Reproduced with permission from *Analytica Chimica Acta*; published by Elsevier, 2018.

Determinations employing luminescence-based detection are much less frequently reported flow-injection methods than those with absorptive spectrophotometric detection, in spite of the wide availability of commercial instrumentation and what are usually much better limits of detection. Several examples of methods using fluorometric detection have been developed in the past decade; these are also listed in Table 2. A particularly low LOD was achieved, e.g., in the SIA determination of ammonia [93], Cd(II), and Pb(II) [94], and in the FIA system for the determination of phosphate [95] and sulfide [86]. In the method first mentioned, with a complex branched manifold (Figure 4), the pressure-assisted dual-headspace gas-liquid micro-extraction step, based on the lab-in-syringe concept, was employed on-line. The developed analytical procedure involved the in situ generation of ammonia vapor for its further dissolution and the formation of a fluorescence product. A sampling rate of 8 h^−1^ was reported for very sensitive determinations with an LOD of 50 ng L^−1^.

Chemiluminescence detection, based on the principle of the permanganate method, was used for the determination of chemical oxygen demand in an FIA system [97], as well as for the very sensitive determination of Hg(II) in surface- and tap waters [98]. In the latter example, an immunoassay based on the use of resin beads, enzymatic amplification, and a novel monoclonal antibody with strong affinity recognition and high specificity for Hg(II), were used.

Yet another application of molecular spectroscopy in the UV-Vis range is the use of thermal lens spectroscopy in an FIA system for the sensitive determination of Ag(I), based on the formation of colloidal silver nanoparticles [99]. This detection is based on the measurement of the temperature rise of an illuminated sample and the index of refraction modification as a result of nonradiative relaxation of the energy absorbed by the analyte from a laser.

Flow-injection systems with on-line sample-processing operations, employed mostly for analyte preconcentration or the elimination of interfering matrix components, are also combined with atomic spectroscopy instruments [120,121,122,123,124,125,126,127,128,129,130,131,132,133,134,135]. They are commonly used for determining numerous trace elements in water analysis (Table 3). In this case, the absorptive measurements are also most frequently exploited using different atomization techniques. Spectrometry with flame atomization was used in the FIA determination of Cd(II) [120], and Cr(III) [121], in both cases using on-line analyte enrichment with ion-imprinted polymers, with appropriate cavities produced during the polymerization for the selective uptake of the given ions. Determinations of Cd(II) were carried out, with preconcentration on magnetic ligand-functionalized nanoparticles [122], while for the determination of Cd, Co, and Cu, an SPE with ligand-functionalized silica gel was reported [123]. The original attempt at the use of flame-AAS detection is the indirect determination of sulfite, based on the on-line reduction of MnO_2_ and the measurement of quantitatively produced Mn(II) [124].

**Table 3:** The application of atomic spectroscopy detection methods in flow-injection systems for water analysis.

Analyte(s)	Type of Water	Detection Method	Type of Flow System	Employed Sample Processing/Remarks	LOD, mg L^−1^	1st Author, Year of Publ.	Reference
As, inorganic	Mineral, tap, river waters	HG-AAS	SIA	SIA system equipped with on-line gas-liquid separator/reaction chamber and electrothermal quartz flow through atomizer	50 ng L^−1^	Anthemidis, 2014	[127]
As, inorganic	Tap, ell and sea waters	HG-AFS	FIA	FIA system equipped with microcolumn with immobilized tetrahydroborate and gas-liquid separator	13 ng L^−1^ for As(III)	Wang, 2014	[133]
Bi, Hg, Sb, Sn	Lake waters	CVG-ET- AAS	FIA	Sequential determination of analytes using a high-resolution continuum source AASD and on-line SPE for separation and preconcentration	Bi 1, Hg 170, Sb 9, Sn 180 ng L^−1^	Guerrero, 2015	[130]
Cd(II)	Ground and wastewater	Flame AAS	FIA	Cd(II)-imprinted polymer employed for on-line preconcentration	0.00011	Gawin, 2010	[120]
Cd(II)	Ground and sea waters	CV-AAS	Multisyringe FIA	System with on-line gas-liquid separator	5.8 ng L^−1^	Silva, 2014	[129]
Cd(II)	Surface waters	Flame AAS	FIA	On-line preconcentration using microcolumn with renewable magnetic ligand-functionalized nanoparticles	0.002	Rocha, 2021	[122]
Cd(II), Co(II), Cu(II)	Water standard	Flame AAS	FIA	On-line preconcentration using microcolumn with ligand-functionalized silica gel	N.a.	Sivrikaya, 2018	[123]
Co(II)	Ground and tap water	ET-AAS	LOV-SIA	On-line SPE extraction and preconcentration using functionalized magnetic nanoparticles	6 ng L^−1^	Wang, 2012	[125]
Cr(III)	Municipal wastewater	Flame AAS	FIA	Cr(III) imprinted polymer for on-line separation and preconcentration	0.0021	Leśniewska, 2015	[121]
Hg speciation	Lake water	AFS	SIA	Speciation of inorganic and organic mercury forms	3 ng L^−1^	Zhang, 2018	[134]
Pb(II)	Lake, sea, river waters	HG-AAS	FIA	On-line SPE preconcentration ligand-functionalized sorbent	82 ng L^−1^	Trujillo, 2013	[128]
Noble metals	Lake, tap and sea waters	ICP-AES	FIA	Simultaneous determination of Pt, Pd, Os, Ir, Rh, Ag and Au employing magnetic ligand-functionalized nanoparticles for on line SPE	Ag 0.03, Pd 1.5, Rh 100 mg L^−1^	Guerrero, 2017	[131]
Sb speciation	Surface, tap, ground water	HG-AFS	Multisyringe FIA	FIA system with cationic minicolumn for retaining trimethylantimony and gas-liquid separator	Sb(III), (V) 30 TMSb 130 ng L^−1^	Portugal, 2015	[135]
Se(IV), Se(VI)	Tap water	HG-AAS	FIA	Ag nanoparticles employed for catalytic activation in Se(IV) determination	0.0005	Poonyaka 2017	[126]
Sulfite	Mineral, tap, river waters	Flame AAS	FIA	On-line reduction in a microcolumn packed with MnO_2_	0.08	Zare- Dorabei, 2018	[124]
TOC	River waters	AES	CFA	Flow system including miniaturized dielectric barrier carbon AES with on-line microwave-assisted oxidation	0.01 as C	Han, 2014	[132]

Abbreviations used: AAS—atomic absorption spectrometry, AES—atomic emission spectrometry, CFA—continuous flow analysis, CV-AAS—cold vapor atomic absorption spectrometry, FIA—flow injection analysis, HG-AAS—hydride generation atomic absorption spectrometry, HG-AFS—hydride generation atomic fluorescence spectrometry, ICP—inductively coupled plasma, SIA –sequential injection analysis, SPE—solid-phase extraction, TOC—total organic carbon. N.a.—no information available.

**Figure 5:**
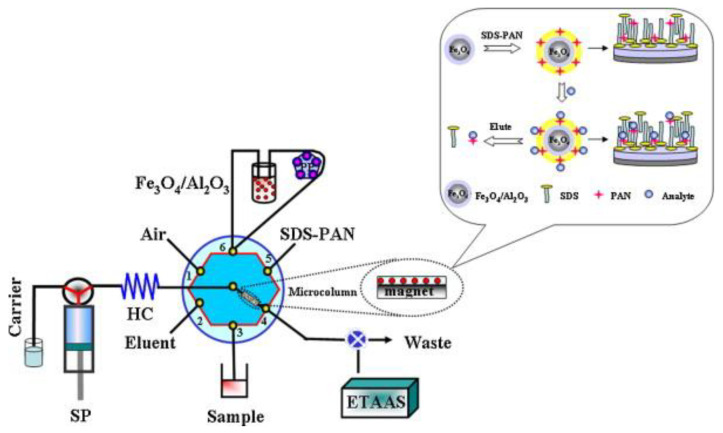
Schematic diagram of an SIA system incorporating a lab-on valve for the trace determination of cobalt in tap- and groundwater, with electrothermal-AAS detection [125]. SP, syringe pump; carrier, distilled water; HC, holding coil; eluent, ethanol; PP, peristaltic pump; ETAAS, electrothermal atomic absorption spectrometer. Reproduced with permission from *Analytica Chimica Acta*; published by Elsevier, 2012.

Rather sporadically, one can also find examples of the hyphenation of an SIA system with an AAS instrument operating with electrothermal atomization (see, e.g., the schematic diagram of a manifold in Figure 5 [125]). With the use of on-line preconcentration on alumina-coated iron oxide nanoparticles that were functionalized with sodium dodecyl sulfate, cobalt (II) was determined in water samples in an SIA-LOV system connected on-line to an AAS spectrometer, with an impressive LOD of 6 ng L^−1^.

**Figure 6:**
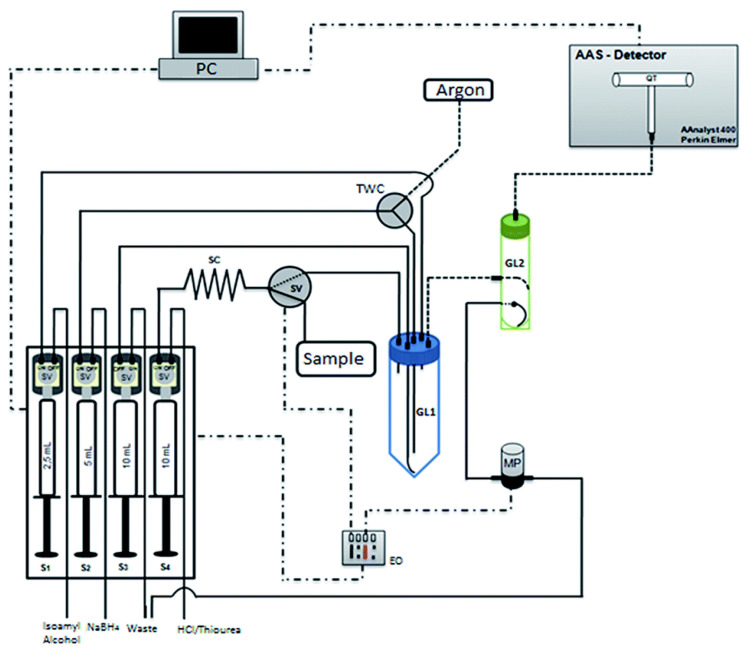
Schematic diagram of the multisyrimge FIA system developed for trace determination of cadmium in ground and sea waters using cold vapor-AAS detection [129]. GL—gas–liquid separator; MP—micro-pump; QT—quartz tube; SC: sample coil; SV—solenoid valve, and TWC—three-way connector. Reproduced with permission from *Journal of Atomic Absorption Spectrometry*; published by the Royal Society of Chemistry, 2014.

On the other hand, numerous flow-injection systems were reported that employed hydride generation. They were developed, for instance, for the speciation analysis of Se [126], and for very sensitive determinations of inorganic arsenic [127] and Pb(II) [128]. The cold vapor-AAS technique is used mostly in the trace determination of mercury, but in one work on a multisyringe FIA system, CV-AAS detection was employed in the determination of Cd(II) in ground- and seawater. A very low LOD of 5.8 ng L^−1^ was obtained without an additional preconcentration step, but with the addition of thiourea to increase the analytical signal [129]. A schematic diagram of the developed manifold is shown in Figure 6.

Then, in the determination of Bi and Pb with similar detectability, chemical vapor generation and an electrothermal-AAS with a continuum source of radiation were used, together with an on-line SPE on ligand-functionalized sorbent [130]. In the same setup configuration, the limits of detection for Hg and Sn were much poorer.

**Figure 7:**
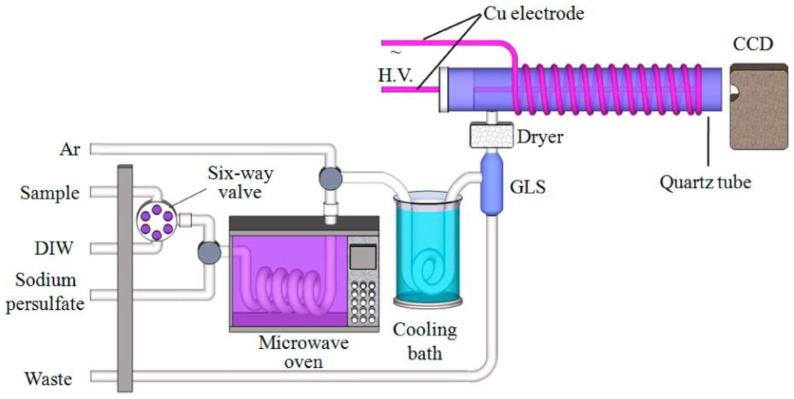
Schematic diagram of a flow system with on-line microwave-assisted oxidation and atomic spectrometry detection, based on the use of miniaturized dielectric barrier carbon developed for the determination of total organic carbon in river waters [132]. GLS—gas-liquid separator; CCD—charge-coupled device spectrometer. Reproduced with permission from *Analytical Chemistry*; published by the American Chemical Society, 2014.

The chemical vapor generation of analytes prior to ICP-OAS detection allows the introduction of a larger sample to increase sensitivity, and, with the additional preconcentration of analytes on the magnetic nanoparticles functionalized with ligands, this was used in an FIA system for the simultaneous determination of several noble metals in natural waters [131]. Another mode of atomic spectroscopy detection has been employed very recently for the determination of total organic carbon (Figure 7). In this case, detection was based on the use of miniaturized dielectric barrier discharge carbon atomic emission, with the microwave-assisted oxidation of organic analytes [132]. The LOD for these determinations was evaluated at 10 μg L^−1^ C, and the developed system can also be used for in situ monitoring in routine applications.

Very low limits of detection were also reported for an FIA system with hydride generation-atomic fluorescence spectrometry (AFS) for the determination of inorganic arsenic [133]. Flow-injection systems with AFS detection were also reported for the determination of mercury [134] and antimony [135].

### 3.2. Application of Electrochemical Detection

Although certain electroanalytical techniques find many common applications in routine laboratories for water analysis, such as potentiometry with membrane ion-selective electrodes (ISE) or voltammetry for trace metal analysis, the past decade did not bring too many examples of the application of those techniques in FIA systems for water analysis [136,137,138,139,140,141,142,143,144] (Table 4). The developed flow-injection systems dealt only with the determination of inorganic analytes, and the reported limits of detections very seldom competed with those obtained via the use of spectroscopic detection. An LOD level of ng L^−1^ for the determination of Hg(II) in river water was reached in an FIA system, with the use of anodic stripping voltammetry (ASV) with gold or platinum working electrodes, wherein metals were sputtered onto microporous membranes [136]. Then, detection with square wave voltammetry (SWV) was employed in the determination of phosphate in an SIA system, in which a glassy carbon electrode, modified with silanized carbon nanotubes and Au nanoparticles, was used as the working electrode [137]. The employed modification of the electrode surface enabled the researchers to obtain an increase in the catalytic response current of a molybdophosphate complex. A similar modification using the nanoparticles of a carbon screen-printed electrode was also reported in an FIA system with amperometric detection for the selective determination of As(III) in tap water and industrial wastewaters [138].

**Table 4:** Examples of the application of electroanalytical detection methods in flow-injection systems for water analysis.

Analyte(s)	Type of Water	Detection Method	Type of Flow System	Employed Sample Processing/Remarks	LOD, mg L^−1^	1st Author, Year of Publ.	Referen- ce
Ammonium, sulfide	City canal water	Conduc-tivity	FIA-SIA	Hyphenation of FIA systems with membraneless vaporization units with SIA systems for contactless conductivity detection	NH_4_^+^ 2.0 S^2−^ 1.9 μM	Alahmad, 2018	[142]
Ammonium, TIC	Tap, well seawaters	Conduc-tivity	FIA	A solenoid micropumping FIA system with a gas-diffusion module for CO_2_ and NH_3_ separation	NH_4_^+^ 0.27 TIC 50 μM	Henriquez, 2014	[141]
As(III)	Tap and industrial wastewater	Ampero- metry	FIA	A carbon screen printed electrode modified with a gold nanoparticle/carbon nanofiber/chitosan	0.0114	Nellaiappan, 2018	[138]
Ca, K, Na, chloride	CRM of different waters	Potentio- metry	FIA	3D-printed multi-electrode flow through cell incorporating plasticized PVC-based ion-selective electrodes	N.A.	Dębosz, 2020	[139]
Carbonate, NH_3_-N, salinity	River, tap, seawater	Conduct- ivity	FIA	An FIA system incorporating membrane units for CO_2_ and NH_3_ separation and a dual-channel contactless conductivity detector	CO_3_ 0.31 mM N 1.85 μM Salinity 0.24%	Chaneam, 2018	[140]
Cr(VI)	Mineral water	Capacitance	FIA	Detection based on the electrostriction phenomenon, observed with a thiol modified gold electrode	4.7 nM	Wieczorek, 2017	[143]
Hg(II)	River water	ASV	CFA and FIA	Au or Pt working electrodes prepared by sputtering of metals onto microporous membranes	CFA 40 FIA 50 ng L^−1^	Mizuguchi, 2013	[136]
Nitrate	River, tap well water	Potentio- metry	SIA	Detection with PVC-based nitrate ISE and sodium ISE used as reference electrode	0.36 μM	Tossanaitada 2012	[144]
Phosphate	Lake and pool water	SWV	SIA	Detection with a glassy carbon electrode, modified with silanized carbon nanotubes and Au nanoparticles	0.3	Wu, 2021	[137]

Abbreviations used: ASV—anodic stripping voltammetry, CRM—certified reference materials, FIA—flow injection analysis, ISE—ion-selective electrode, SIA—sequential injection analysis, SWV—square wave voltammetry, TIC—total inorganic carbon. N.A.—not available.

The application of potentiometric detection in an FIA system was reported for K^+^, Na^+^, Ca^2+^, and Cl^−^ ions commonly occurring in natural waters, for which membrane ISEs are available [139]. In the developed system, a 3D-printed flow cell was used, incorporating indicator electrodes with plasticized membranes; the measuring setup was employed successfully for the analysis of several certified reference materials of different water samples.

**Figure 8:**
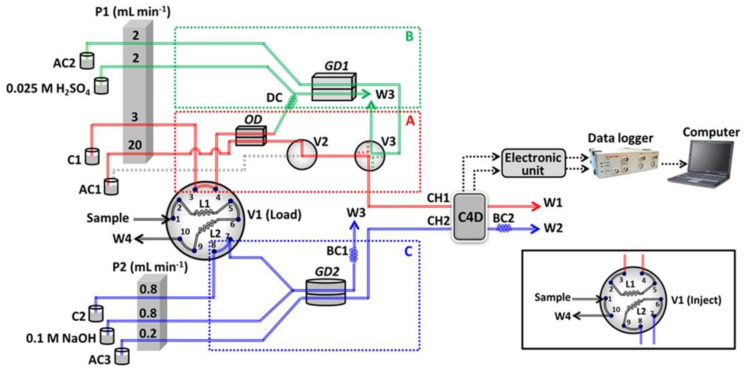
Schematic diagram of an FIA system with conductivity detection, developed for the simultaneous determination of salinity (section A), carbonate (section B), and ammonium nitrogen (section C), using a dual-channel C^4^D detector [140]. P1 and P2—peristaltic pump; L1 and L2—sample loop at 200  µL (L1) and 1000  µL (L2); V1—10-port valve in “load” position, with an inset showing the “inject” position; V2—two-way right-angle switching valve; V—four-way diagonal switching valve; C1 and C2—water carrier; AC1, AC2, and AC3—water acceptor; BC1 and BC2—back-pressure coil (i.d.: 0.75  mm, length: 120  cm); DC—delay coil (i.d.: 1  mm, length: 320  cm); OD—on-line dilution unit; GD1 and GD2—gas diffusion unit; W1–W4—waste; CH1—channel 1 of C^4^D. CH2—channel 2 of C^4^D. Reproduced with permission from *Talanta*; published by Elsevier, 2018.

Detection with a non-selective conductivity technique in flow-injection systems requires the use of a suitable separation step or the isolation of the analyte. In three systems that can be found in the recent literature on water analysis, this detection method was employed for multicomponent determinations [140,141,142]. For instance, in the FIA system of configuration shown schematically in Figure 8, the simultaneous detection of carbonate, salinity, and ammonium nitrogen was achieved [140]. The measuring setup was equipped with a dual-channel contactless C^4^D conductivity detector and two gas diffusion modules, not only for the separation of the gaseous CO_2_ and NH_3_ but also for on-line dilution for salinity determination. Its successful application was described for the analysis of river, sea, and tap waters, with a sampling rate of 20 samples h^−1^, which can be considered very satisfactory. The developed system is able to tolerate most of the matrix constituents present in natural water samples. Another FIA system with conductivity detection has been developed for the simultaneous determination of ammonia and total inorganic carbon [141], as well as for ammonia and sulfide [142], with micromolar limits of detection.

The very sensitive determination of Cr(VI) can be carried out in the FIA system with the use of detection exploiting the electrostriction phenomenon, based on the measurement of membrane capacitance [143]. This phenomenon refers to the effect of a slight change of shape, or mechanical deformation, under the application of an electric field. In the cited work, a gold electrode with a thiol self-assembled monolayer and additional functional groups was used, and the analyte interacted with that dielectric layer.

### 3.3. FIA Systems Using Other Detection Methods

**Figure 9:**
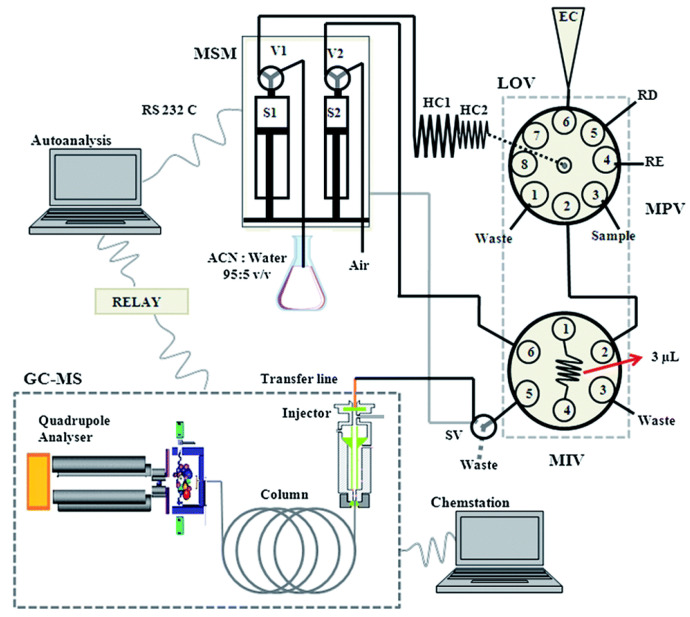
Schematic diagram of the LOV-SIA system developed for on-line sample processing prior to the gas chromatography setup, for the determination of polycyclic hydrocarbons in river, tap, and rain waters [146]. MPV—multiposition valve; MSM—multisyringe module; MIV—micro-injection valve; RD—reagent dispersant (acetonitrile); RE—reagent extractive (trichloroethylene); HC 1–2—holding coils; SV—solenoid valve; S 1–2—syringe pumps; V 1–2—valves; EC—extraction chamber. Reproduced with permission from *Analytical Methods*; published by the Royal Society of Chemistry, 2014.

In this group of flow-injection systems that were developed for water analysis and are listed in Table 5, the application of mass spectrometry predominates. These are not methods based on the interaction of electromagnetic radiation with matter; thus, according to the IUPAC recommendations for terms used in analytical spectroscopy [145], they are not included in Section 3.1. In only one case from the collected works [146,147,148,149,150,151,152,153,154,155,156,157], a flow system was designed for the determination of organic pollutants, namely, the SIA-LOV system, which was hyphenated to gas chromatography (GC) with MS detection [146]. In this complex instrumental configuration, shown schematically in Figure 9, the on-line sample processing was involved with the LOV-based dispersive liquid-liquid extraction carried out in the multisyringe SIA setup. For the determination of 16 priority polycyclic aromatic hydrocarbons in water, using 4 mL of sample volume, and good enrichment factors (27 to 38), the obtained LOD values at ng L^−1^ level were reported.

**Table 5:** Examples of the application of detection methods other than molecular and atomic spectroscopy or electrochemical detection in flow-injection systems for water analysis.

Analyte(s)	Type of Water	Detection Method	Type of Flow System	Employed Sample Processing/Remarks	LOD, mg L^−1^	1st Author, Year of Publication	Reference
Chloride, sulfate	Mineral waters	Piezoelectric	FIA	Detection using a flow-through acoustic sensor with a quartz crystal with a deposited ionophore layer	Cl^−^ 50 μM SO_4_ 42 μM	Venancio, 2018	[155]
COD	Lake and river waters	Thermal sensor	FIA	Determination is based on measuring the heat generated when the sample passes through a column containing the periodic acid solution	1.84	Yao, 2014	[156]
PAHs	River, tap, rain waters	MS	Multisyringe SIA-LOV-GC	SIA system with on-line dispersive liquid-liquid micro-extraction	From 10 to 70 ng L^−1^	Clavijo, 2014	[146]
Pb, Sr	River and rainwater	ICP-MS	Multisyringe SIA-LOV	On-line preconcentration with Sr-resin	Pb 4 Sr 12 ng L^−1^	Beltran, 2015	[151]
Re	Seawater	ICP-MS	FIA	On-line preconcentration on Dowex 1 × 8 anion-exchange resin	0.1 ng L^−1^	Zhu, 2017	[150]
Sr	River water	ICP-MS	SIA-LOV	Application of commercial Sr-resin for off-line separation and preconcentration of radionuclide ^90^Sr in SIA system	2.9 ng L^−1^ 14.5 Bq L^−1^	Kołacińska, 2017	[147]
Tc	Mineral, tap surface	ICP-MS	Multisyringe SIA-LOV	Application of commercial resin TEVA for on-line separation and preconcentration of radionuclide ^99^Tc in SIA system	49 pg L^−1^	Rodriguez, 2015	[149]
Tc	River and wastewater	ICP-MS	SIA-LOV	Application of commercial resins Dowex 1 and TEVA for off-line separation and preconcentration of radionuclide ^99^Tc in SIA system	9.55 pg L^−1^ 6.0mBq L^−1^	Kołacińska, 2018	[148]
Tc	River, sea, groundwater	ICP-MS	FIA	Application of commercial resin TK201 for on-line separation and preconcentration of radionuclide ^99^Tc	9.3 pg L^−1^ 5.9mBq L^−1^	Matsueda, 2021	[157]
Trace metals	Seawater	ICP-MS	FIA-IC	FIA system coupled with ion chromatography with ICP-MS detection and on-line preconcen-tration on chelating resin for determination of Al, Cd, Co, Cr, Fe, Mn, Ni, Pb, Ti, V, Zn	From ~0.03 (Cd) to ~100 (Al) ng L^−1^	Ho, 2010	[154]
Trace metals	Open-ocean water	ICP-MS	FIA	On-line preconcentration on minicolumn packed with a chelating sorbent for determination of Cd, Co, Cu, Ni, Pb, Zn	1.5 (Pb) to 71 (Zn) pM	O’Sullivan, 2013	[152]
Trace metals	Seawater	ICP-MS	FIA	Application of commercial resins for on-line separation and preconcentration of Co, Fe, Pb and V in the flow system	From 0.0058 (Co) to 0.34 (Fe) nM	Clough, 2015	[153]

Abbreviations used: COD—chemical oxygen demand, FIA—flow injection analysis, GC—gas chromatography, IC—ion chromatography, ICP-MS—inductively coupled plasma—mass spectrometry, SIA—sequential injection analysis, LOV—lab-on-valve, MS—mass spectrometry, PAHs—polycyclic aromatic hydrocarbons.

Much more frequently, FIA and SIA systems are being developed for elemental analysis with the use of very sensitive, inductively coupled plasma-mass spectrometry (ICP-MS). The main purpose of combining a very selective ICP-MS detection with flow-injection systems is usually the possibility of the additional elimination of isobaric interference in MS detection, elimination of the interfering effects of sample matrices, and also an additional preconcentration of analytes. These goals can be achieved in an off-line configuration without instrumental hyphenation, which was reported, for instance, for the determination of ^90^Sr [147], and ^99^Tc [148] radionuclides, especially in the second case with SPE preconcentration, where an LOD was achieved that was as low as 9.55 pg L^−1^. For the determination of ^99^Tc, on-line setups were also developed, with an on-line SPE using different commercial sorbents (e.g., [149]). Similar flow systems were described for the trace determination of rhenium in seawater [150], of Pb and Sr in river and rain waters [151], and also for the simultaneous determination of many trace elements in seawater [152,153]. In all those systems, the analytes were preconcentrated on-line on solid sorbents. For a similar purpose, an FIA setup with on-line enrichment can be hooked up to a high-performance ion-chromatograph and ICP-MS spectrometer, which allows sample processing for the simultaneous determination of 12 analytes in seawater, taking about 10 min [154].

Among other detection techniques employed in FIA systems for water analysis, one can find the application of piezoelectric detection [155], as well as thermal sensing [156]. In the first study, the detection method was based on the use of a quartz crystal oscillator attached to an electrode covered with a layer containing the appropriate ionophore for the detection of chloride and sulfate, with micromolar LODs. In the second study, a determination of the chemical oxygen demand in water was based on measuring the heat generated by the reaction of organic analytes with periodic acid, packed into a mini-column.

### 3.4. Microfluidic Flow-Injection Systems Dedicated to Water Analysis

One particular instrumental novelty in the field of water analysis in recent years seems to be an upsurge of interest in the construction of microfluidics, mostly used so far for capillary electrophoretic determinations and for clinical and biochemical determinations. They can be used for flow-injection measurements on a much smaller scale, in terms of the sample volume needed and the consumption of reagents. It must be realized, however, that in the present stage of their development, the microfluidic chip usually replaces the hydraulic part of the flow system, eventually including some integrated miniaturized flow-through detection cells or sample processing module. However, it still needs the typical periphery devices in a common macroscale (sampling units, pumps, detection transducers, control units, etc.). This means that, in fact, it is very far from a realistic micro total analytical system. Obviously, the examples collected in Table 6 [158,159,160,161,162,163,164,165] do not include microfluidic devices involving, e.g., capillary electrophoretic separations, but they do include the reported miniaturized flow-injection setups.

Similar to typical flow-injection systems, in the case of microfluidics, spectroscopic detections are also commonly used. Among the listed works, in only two cases, electrochemical detection was conducted. The potentiometric setup was used with a chip incorporating an ammonium membrane ion-selective electrode, a gas diffusion unit, and a screen-printed reference electrode [158]. A very sensitive determination of the herbicide residues in groundwater was reported, using an amperometric immunoassay carried out with a microfluidic chip and a screen-printed carbon working electrode [159].

**Table 6:** Examples of application of microfluidics in the flow-injection systems for water analysis.

Analyte(s)	Type of Water	Detection Method	Employed Sample Processing/Remarks	LOD, mg L^−1^	1st Author, Year of Publ.	Reference
Ammonia	Lake water	UV/VIS absorption	FIA system with microfabricated electroosmotic pump coupled to a gas-diffusion microchip	0.10	Zhu, 2015	[160]
Ammonium	Treatment plant	Potentio- metric	On-line integration of gas-diffusion step with a flow cell with ammonium ISE and screen-printed reference electrode	0.07	Calvo-Lopez, 2015	[158]
Bacteria	Lake, wastewater	Fluorescence	Enrichment and determination of *Escherichia coli* using an antibody-modified microfluidic chip, and benchtop real-time PCR	6 cls	Dharmasiri, 2010	[166]
Cr(VI)	River water	UV/VIS absorption	Monolithically integrated system based on green tape ceramic technology, involving an LED-based detector	0.05	Alves Segundo 2011	[161]
Cr(III), Cr(VI)	River and tap waters	Fluorescence	Application of the magnetic-carbon nanotube nanocomposite for off-line solid-phase extraction preconcentration	94 pM	Peng, 2017	[165]
Herbicide	Ground- water	Ampero- metric	Immunoassay of 2,6-dichlorobenzamide residues in a microfluidic setup with a screen-printed electrode for amperometric detection	20 ng L^−1^	Uthuppu, 2015	[159]
Iodine, total inorganic	Ground- water	Fluorescence	Determination based on the use of the Sandell–Kolthoff reaction and fluorimetric detection of Cr(III)	0.0077	Inpota, 2018	[164]
Nitrite	River water	UV/VIS absorption	Microfluidic system with fluid manipulation using a biomimetic photoresponsive ionogel microvalve	0.034	Czugala, 2013	[162]
Nitrate	Tap, sea and surface waters	UV/VIS absorption	Integrated nitrate analyzer with microfluidic and LED light source with a photodiode detector	0.70	Cogan, 2015	[163]

Cls—colony-forming units.

Numerous interesting innovations have been reported in the design of microfluidics with spectrophotometric detection. For the determination of ammonia, a gas-diffusion microchip was coupled with a microfabricated electroosmotic pump, to perform FIA measurements [160]. The microfluidic device was produced with the use of green tape ceramic technology for the determination of Cr(VI) in river water, with an LOD of 50 μg L^−1^ [161]. Microfluidic chips were also designed for the determination of nitrite [162] and nitrate [163] in water. In the first study, the manipulation of solutions was carried out by the use of a biomimetic photoresponsive ionogel microvalve, controlled by a white light-emitting diode [162].

Several examples in the literature over the last decade can also be found for the application of fluorimetric detection in flow-injection microfluidics. A very sensitive (the LOD 7.7 μg L^−1^) determination of total inorganic iodine was reported, using a microfluidic chip, based on the reaction between Ce(IV) and As(III), catalyzed by iodide [164]. The determination of Cr(III) and Cr(VI) with on-line fluorescent derivatization was conducted in a dedicated microfluidic chip, following off-line solid-phase extraction using magnetic carbon nanotubes [165]. A microfluidic chip with immobilized antibodies was employed prior to performing the determination of bacteria via an off-line benchtop real-time quantitative polymerase chain reaction.

## 4. Portable Flow-Injection Instrumentation for Water Analysis

Water monitoring requires not only the development of efficient analytical methods for use in specialized laboratories but also portable instrumentation with remote control for field applications. Such an apparatus is often needed for carrying out process analysis at technological installations (e.g., for the continuous monitoring of processing in wastewater treatment plants), as well as for in-field applications related to environmental monitoring, such as the control of pollution in river waters, or for monitoring natural processes occurring in seas and oceans. In the design of such portable instruments, since the early 1980s, a flow-injection methodology has been exploited (see, e.g., review [167]). The construction of such devices is based on the suitable choice and integration of a propulsion system, sampling and injection tools, and selection of the most satisfactory detection technique, depending on the target analyte(s) and the required selectivity. Such a system should be computer-controlled (see, for instance, [168]), although in many of the works cited in this review that include the use of commercial equipment, the flow-injection systems that are employed are fully computerized.

The analytical literature on the development of flow-injection methods and instrumentation from the 1980s provides numerous examples of such portable systems constructed for water analysis. Several such systems have also been developed in the past decade. The FIA system for monitoring total organic carbon has already been mentioned above [132]. It is based on the use of carbon atomic emission spectrometry for detection, as well as sample processing using the on-line microwave-assisted oxidation of analytes by persulfate. The obtained LOD of 10 μg L^−1^ is satisfactory for various environmental applications.

**Figure 10:**
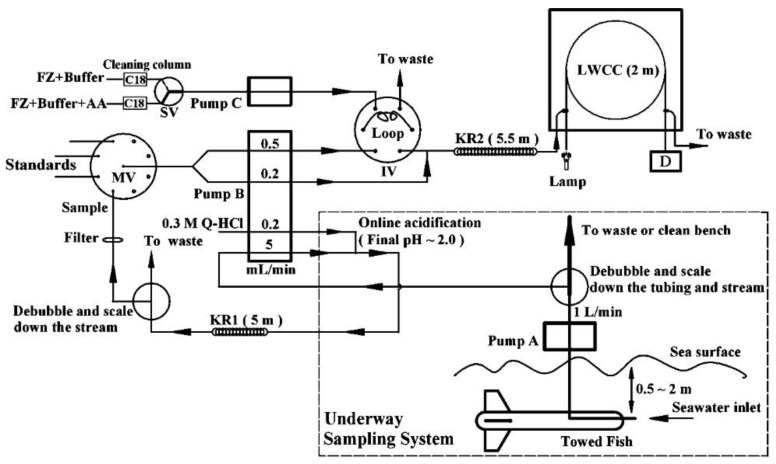
Manifold of the reversed FIA setup for a real-time iron speciation shipboard-use system [171]. The dashed box indicates the towed fish-based underway-sampling system, through which unit surface seawater was pumped continuously onboard, with pump A, at a flow rate of about 1 L min^−1^. MV, IV, and SV are the multiposition valve, six-port injection valve, and three-way solenoid switching valve; KR is the knitted reaction coil; FZ and AA are ferrozine and ascorbic acid. Reproduced with permission from *Environmental Science and Technology*; published by the American Chemical Society, 2015.

In the past several decades, numerous measuring systems have already been designed for submersible measurement, as well as setups for shipboard measurement on research vessels, with underway-sampling probes. In recent years, several such units have been developed by a research group from the State Key Laboratory of Marine Environmental Science in Xiamen University, Xiamen, China [169,170,171,172,173]. These were developed using absorptive spectrophotometric detection to identify trace dissolved manganese in estuarine and coastal waters [169], for the determination of dissolved sulfide [170], and for the redox speciation of iron, with sub-nanomolar LODs [171]. The configuration of the manifold of the latter study is shown in Figure 10. This reverse-FIA setup employs a liquid waveguide capillary flow cell, and the detection of Fe(II) is based on a reaction with ferrozine, while Fe(III) was reduced by ascorbate. In two other systems that have been mentioned [169,170], reverse-FIA systems were also used, with reported nanomolar limits of detection. The ultra-trace on-board determination of ammonium was based on the use of the fluorimetric detection of the reaction product of ammonia with *o*-phthalaldehyde [172,173]. In a flow-batch system via the SPE preconcentration of the fluorescent product, an LOD of 1.2 nM was achieved [172]. The developed systems were successfully used for the determination of ammonia in seawater [172], in estuarine, and in coastal waters [173].

One very challenging attempt was also reported on the design of a potentiometric microsystem for the determination of nitrate and potassium when monitoring a water recycling process, oriented toward its use in manned space missions [174]. It integrates a ceramic microfluidic chip, with two solid-contact polymeric ion-selective electrodes and a screen-printed reference electrode. LODs of below 1 mg L^−1^ were reported for both analytes, which is satisfactory for the given application. A further perspective in the design of that type of setup involves exchanging the conventional pumps and valves for miniaturized, fully integrated devices. A similar system was also reported for the determination of ammonium [158].

## 5. Conclusions

Flow-injection analytical methods, which have been developed for almost half a century, find a wide range of applications in water analysis, usually due to the large sampling rate, the possibility of the application of different detection techniques, and better precision compared to similar manual procedures. The on-line conducting of various sample processing operations is especially attractive and, in flow-injection systems, does not need to be carried out until a steady state of equilibrium is reached, without any loss of precision. These features mean that with an appropriate construction of the measuring system, they can also be used in remote and portable configurations, and they may also serve as early warning systems in processing or environmental applications [68].

In a further improvement of those methods. Novel sorption materials are being introduced, along with new methodologies for undertaking various sample-processing operations. The primary directions of further development seem to be the miniaturization of measuring setups, with the integration of various modules, the introduction of elements of real automation, especially those involving the optimization of measuring conditions without human effort, and the further popularization of those methods as a convenient tool for sampling and sample processing, prior to high-performance separations using chromatographic and electrophoretic methods.

## Data Availability

Not Applicable.
